# Stable Isotope Evidence for Dietary Overlap between Alien and Native Gastropods in Coastal Lakes of Northern KwaZulu-Natal, South Africa

**DOI:** 10.1371/journal.pone.0031897

**Published:** 2012-02-21

**Authors:** Nelson A. F. Miranda, Renzo Perissinotto

**Affiliations:** School of Biological and Conservation Sciences, University of KwaZulu-Natal, Durban, South Africa; Biodiversity Insitute of Ontario - University of Guelph, Canada

## Abstract

**Background:**

*Tarebia granifera* (Lamarck, 1822) is originally from South-East Asia, but has been introduced and become invasive in many tropical and subtropical parts of the world. In South Africa, *T. granifera* is rapidly invading an increasing number of coastal lakes and estuaries, often reaching very high population densities and dominating shallow water benthic invertebrate assemblages. An assessment of the feeding dynamics of *T. granifera* has raised questions about potential ecological impacts, specifically in terms of its dietary overlap with native gastropods.

**Methodology/Principal Findings:**

A stable isotope mixing model was used together with gut content analysis to estimate the diet of *T. granifera* and native gastropod populations in three different coastal lakes. Population density, available biomass of food and salinity were measured along transects placed over *T. granifera* patches. An index of isotopic (stable isotopes) dietary overlap (IDO, %) aided in interpreting interactions between gastropods. The diet of *T. granifera* was variable, including contributions from microphytobenthos, filamentous algae (*Cladophora* sp.), detritus and sedimentary organic matter. IDO was significant (>60%) between *T. granifera* and each of the following gastropods: *Haminoea natalensis* (Krauss, 1848), *Bulinus natalensis* (Küster, 1841) and *Melanoides tuberculata* (Müller, 1774). However, food did not appear to be limiting. Salinity influenced gastropod spatial overlap. *Tarebia granifera* may only displace native gastropods, such as *Assiminea* cf. *ovata* (Krauss, 1848), under salinity conditions below 20. Ecosystem-level impacts are also discussed.

**Conclusion/Significance:**

The generalist diet of *T. granifera* may certainly contribute to its successful establishment. However, although competition for resources may take place under certain salinity conditions and if food is limiting, there appear to be other mechanisms at work, through which *T. granifera* displaces native gastropods. Complementary stable isotope and gut content analysis can provide helpful ecological insights, contributing to monitoring efforts and guiding further invasive species research.

## Introduction

Alien invasive species (AIS) can cause disruptions to ecosystems. However, quantifying their impacts is problematic due to the complexity of ecological interactions [1], [2]. Stable isotope analysis is a powerful quantitative tool for detecting and tracking changes in trophic structure and ecosystem processes (energy flows) caused by abiotic and biotic interactions [3]. The combination of stable isotopes with other techniques is very useful for assessing interactions between several organisms and contributes towards a better understanding of how an ecosystem can be affected by native and non-native species [4], [5]. Stable isotope analysis provides a time-integrated view of ecological processes, whereas gut content analysis reveals snapshots of feeding activity [6]. Stable isotope and gut content analyses are complementary, provide insight into diets and trophic dynamics in ecosystems [7], [8] and have successfully been used in several studies addressing the impacts of AIS on aquatic ecosystems [8]–[12]. Although comparable studies on gastropods are lacking, numerous studies have been published on invasive gastropods such as *Pomacea canaliculata* (Lamarck, 1822) (prosobranch, Ampullariidae) [13]–[15] and *Potamopyrgus antipodarum* (Gray, 1843) (prosobranch, Hydrobiidae) [16], [17], some of which involved stable isotope techniques [18], [19].

*Tarebia granifera* (Lamarck, 1822) (prosobranch, Thiaridae) is a non-native invasive gastropod originally from South-East Asia. This AIS is reported from many tropical and sub-tropical areas of North and South America and Africa [20]–[24]. *Tarebia granifera* was accidentally introduced in South Africa, probably in the 1990s via the aquarium trade [24]. This is reported as one of the most recent introductions of non-native gastropod into South African natural environments, where it has quickly become invasive and widespread, particularly in the KwaZulu-Natal and Mpumalanga provinces [24], [25]. *Tarebia granifera* is parthenogenetic and ovoviviparous, giving birth to live juveniles and often reaching population densities of over 1000 ind. m^−2^ [26]. *Tarebia granifera* has been successfully used as a biological control of schistosomiasis in the Caribbean, since it is able to displace host native gastropods [27]–[29]. In South Africa, *T. granifera* is rapidly invading an increasing number of coastal lakes and estuaries, so there is concern about potential ecological disturbances due to the displacement of native invertebrates [24], [25], [30]. *Tarebia granifera* can have a very high feeding impact and may out-compete native gastropods for food resources [31]. However, the mechanisms through which *T. granifera* may compete with native invertebrates are not yet clear, and questions about its diet still need to be addressed [31].

This study aims to: (1) use gut content and stable isotope analyses to estimate the diets of *T. granifera* and other dominant gastropod populations; and (2) estimate dietary niche overlap between invasive *T. granifera* and native gastropods. The framework of this field-based study also assesses spatial overlap between gastropods as well as availability of food in the form of benthic microalgae. The potential of *T. granifera* to affect trophic dynamics, and compete with native gastropods for food resources, is discussed in light of the findings.

## Materials and Methods

### Ethics Statement

All necessary permits were obtained from the iSimangaliso Wetland Park Authority for the described field studies at each location, under a Research Agreement for the project titled “Climate Change and the Management of KZN estuaries: St Lucia Estuary”.

### Study site

**Figure 1:**
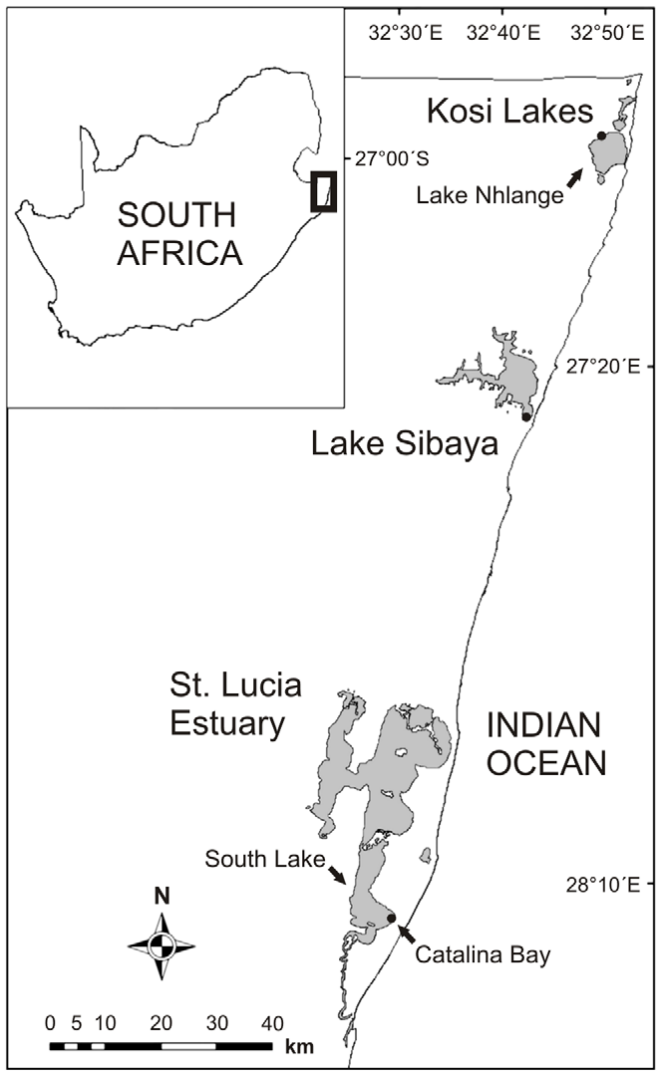
Study area. The Kosi Lakes, Lake Sibaya and the St. Lucia Estuary are Ramsar Wetlands of International Importance within the iSimangaliso Wetland Park, a UNESCO World Heritage Site in Maputaland, northern KwaZulu-Natal, South Africa. Sites sampled are marked as dots on the map.

Sampling was conducted at three different coastal lakes which were all invaded by *Tarebia granifera* over the last decade (Fig. 1).

The St. Lucia Estuary was sampled in June 2007, October 2009 and February 2010. It is the largest estuarine lake in Africa, with a surface area of about 325 km^2^ and average depth of 0.9 m [32]. The sample site, Catalina Bay, is a large limestone flat covered by shallow water and is located on the eastern shores of South Lake (28°13′S, 032°29′E). Vegetation found by the shore included *Cyperus laevigatus*, *Juncus kraussii* and *Salicornia* spp.. Throughout the sampling period, Catalina Bay experienced significant changes in salinity and water level. These were underlined by an unusual mouth breaching event, caused by extreme wave action in the Indian Ocean during March 2007, which resulted in six months of open mouth conditions [33]. Prior to this, the estuary had been completely isolated from the ocean for almost five years.

*Tarebia granifera* has persisted in the St. Lucia Estuary at least since 2005, but its invasion is restricted to freshwater seepage areas along the eastern shores of the South Lake, where it tends to dominate benthic assemblages. Shallow water benthic assemblages are otherwise dominated by the native gastropods *Assiminea* cf. *ovata* and *Haminoea natalensis*. *Assiminea* cf. *ovata* (Krauss, 1848) (previously known as *A. bifasciata*) (prosobranch, Assimineidae) is a small (shell height ≈5 mm) native gastropod with a very wide salinity tolerance that can be found in river mouths, estuaries and lagoons on the east coast of South Africa and Mozambique [34]. *Assiminea* cf. *ovata* is thus well adapted to the hydrological dynamics of the St. Lucia Estuary [35], [36]. *Haminoea natalensis* (Krauss, 1848) (opistobranch, Haminoeidae) occurs from Port St Johns (Eastern Cape) to southern Mozambique [37] and periodically dominates the benthic assemblage along the shallow saline shores of South Lake (pers. obs.).

Lake Sibaya's south-east basin (27°25′S, 032°41′E) was sampled in November 2009 (Fig. 1). This freshwater lake has a surface area of 60 to 77 km^2^ and an average depth of 13 m [38]. It was connected to the ocean in the past and has a number of originally marine and estuarine species uniquely adapted to freshwater conditions [39]. The profile of the littoral zone is steep and, at the time of the study, the level of the lake was low according to previous records [40]. The substrate was composed of white sand and vegetation at the water edge included *Phragmites* sp., *Typha latifolia* and *Cyperus* sp.. Submerged macrophytes, such as *Potamogeton* spp. were dominant close to shore. *Tarebia granifera* dominated the benthic community in the relatively small shallow terrace, where shelter from wave action was provided by vegetation. It is unclear when *T. granifera* was introduced, but its invasion has spread at least along the entire eastern shallow shores of Lake Sibaya. The native *Bulinus natalensis* (Küster, 1841) (pulmonate, Planorbidae) was mostly found on submerged macrophytes. *Bulinus natalensis* occurs on the lowlands of KwaZulu-Natal, South Africa [41].

Kosi Lakes' Lake Nhlange (26°57′S, 032°49′E) was sampled in November 2008 (Fig. 1). The lake has a surface area of 30.7 to 37 km^2^ and an average depth of 7.2 m [42]. Lake Nhlange experiences freshwater conditions, despite being part of a system that is connected to the Indian Ocean [42]. The substrate was composed of clear white sand and marginal vegetation included *Phragmites* sp, *Cyperus* sp. and *Juncus kraussii*. Submerged macrophytes *Ceratophyllum demersum* and *Nymphaea* spp. were found at the water's edge. The benthic assemblage in the sampling area was once again dominated by *T. granifera*, with a few *Melanoides tuberculata* individuals also found occasionally. *Tarebia granifera* was found at boat launching areas where it was noticed only in recent years. *Melanoides tuberculata* (Müller, 1774) (prosobranch, Thiaridae) is composed of a great variety of lineages or morphs which are conchologically and genetically distinct [22]. *Melanoides tuberculata* has a worldwide natural distribution, including tropical Africa, Asia and Oceania. An alien morph of *M. tuberculata* from south-east Asia has invaded Africa [43]–[45]. Although it is assumed that the *M. tuberculata* in this study is native, this remains to be confirmed.

### Transects

*Tarebia granifera* populations tended to be concentrated in dense patches. To assess spatial overlap between *T. granifera* and other gastropods, 4 point transects were placed parallel to the shore line and across patches of *T. granifera*. Transects were selected where *T. granifera* overlapped with a single native gastropod species. Transect lengths varied depending on the area covered by the *T. granifera* patch. Points were placed at even spaces so that points 1 and 4 were at the edge of the *T. granifera* patch and points 2 and 3 were within the patch. Samples were collected in triplicate with a Zabalocki-type Ekman grab (sampling area 0.0236 m^2^) and fixed in 10% formalin. In the laboratory, gastropods were counted and densities (ind. m^−2^) were calculated. Shell heights (SH) were measured with Vernier calipers. Salinity was measured with a YSI 6920 multiprobe.

To estimate microphytobenthos (MPB) biomass, duplicate sediment cores were collected (upper cm only) with a Perspex corer (internal diameter: 20 mm) at every point along transects. Chlorophyll *a* and phaeopigments were extracted in 90% acetone at 4°C over a 48 h period and then measured using a 10-AU Turner Designs fluorometer fitted with a narrow-band, non-acidification system [46]. Chlorophyll *a* and phaeopigment concentrations are added and reported as pigment concentration (mg. m^−2^) [47].

### Gastropod gut content analysis

Twenty individuals from each species were randomly collected along each transect (see above) and immediately preserved in 10% formalin to prevent further digestion [48]. The digestive tract was later removed and the gut contents were extracted with a fine pipette under a dissecting microscope (40× magnification). Gut contents were then viewed under an inverted microscope (400× magnification) and classified based on gross morphology [49]. The following classes were used: microalgae, detritus, filamentous algae, and sand particles.

### Stable isotope samples

Gastropods and their potential food sources were collected and stored separately, first in a cooler box and later in a freezer at −20°C, before being processed and analysed.

Gastropods were collected at random along each transect (see above). Muscle tissue from the foot of individual snails was dissected and pooled to create representative composites. Five replicates were prepared for each species, containing either 2 or 10 individuals each (depending on size).

Potential types of food sources for gastropods were sampled in triplicate along each transect. Reeds, sedges, grasses and macrophytes as well as filamentous algae (*Cladophora* sp.) and detritus (DTR) were collected and thoroughly cleaned in distilled water to remove sediment particles and organisms. These samples were then independently homogenized and freeze dried with a mortar and pestle and liquid nitrogen. Sediment cores were collected with a Perspex corer (diameter 7 cm). The upper cm layer of sediment was suspended in a 2 L container of filtered water and stirred, so that microphytobenthos (MPB) stayed in suspension while sediment and sedimentary organic matter (SOM) settled to the bottom. The sediment was rinsed thoroughly with distilled water, treated with 1 M hydrochloric acid (HCl) for 24 h to remove carbonates, rinsed with distilled water, and then homogenized and freeze-dried as described above. The supernatant containing MPB was collected on a pre-combusted GF/F filter. MPB samples were treated with excess 2% HCl over 2 h, rinsed with distilled water, dried in an air-circulating oven at 60°C for 24 h and wrapped in tin foil prior to being sent for isotopic analysis. All other samples were weighted to appropriate quantities for analysis (5×8 mm tin capsules with approximately 1 mg sample or 20 mg sample in Eppendorf microcentrifuge tubes in the case of sediment).

### Stable isotope analysis

Carbon and nitrogen stable isotope ratios and C/N values were measured at the Stable Light Isotope Unit of the Department of Archaeology, University of Cape Town, South Africa. A Flash EA 1112 series elemental analyzer (Thermo Finnigan, Italy) was used with a Delta Plus XP IRMS (isotope ratio mass spectrometer) (Thermo electron, Germany) and a Conflo III gas control unit (Thermo Finnigan, Germany). Isotopic ratios were expressed as δ values (‰) relative to the Vienna PeeDee Belemnite standard for carbon and to atmospheric N_2_ standard for nitrogen according to:
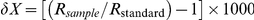
Where *X* is ^13^C or ^15^N and *R* is the corresponding ratio of ^13^C/^12^C or ^15^N/^14^N. A lipid normalization procedure was not followed [50], since a mathematical normalization (e.g. [51]) did not significantly affect the major outcomes of these analyses.

Highest average trophic position (*TP*) of gastropods was calculated with the following equation (adapted in [4]):
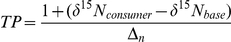
where δ^15^N_consumer_ is the average δ^15^N signature measured directly from a gastropod species, δ^15^N_base_ is the average δ^15^N signature of the most δ^15^N depleted food source, and Δ*_n_* is the assumed average enrichment in δ^15^N per trophic level (Δ*_n_* = 2).

### Data analysis

The Bayesian isotopic mixing model SIAR v 4.0 (Stable Isotope Analysis in R, [52]) was used to generate probability function distributions, showing the most feasible solutions to the contribution of different types of potential food sources to the diet of gastropods, with 95%, 75% and 25% credible intervals. Fractionation correction values of 0.4‰ δ^13^C and 2‰ δ^15^N were assumed [53]. Preliminary analyses were run with the aim to determine the appropriate number of sources in the mixing models [54], [55]. Based on results from gut content analyses and mixing model trial runs, a narrower set of sources was selected for each gastropod species, which provided a better resolution of results [56].Sources that were not found in the gut content analysis and/or had minor contributions (<5%) in the trial mixing models were omitted from the final mixing models. Isotopic dietary overlap (IDO, %) between species *j* and *k* was then calculated with the following equation based on Schoener's Index [57], [58]:
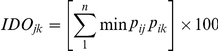
where *p* is the SIAR mean contribution of source *i* resulting from the final mixing models run for subjects *j* and *k* respectively, and *n* is the total number of different resources used by both *j* and *k*. IDO values have an absolute limit of 100%, which indicates complete overlap, and values exceeding 60% were considered to indicate significant dietary overlap [57].

Normality and equality of variance of stable isotope data were confirmed, and analysis of variance (ANOVA) was used for comparisons between locations and times. Pearson correlation was used to assess relationships between availability of food, gastropod abundance and diet. A t-test was used to compare trophic position between *T. granifera* and native gastropods. A linear regression was used to assess the relationship between gut content and stable isotope results. Analyses were done with the statistical package SPSS version 19 for Windows.

## Results

### Spatial overlap and resource availability

**Figure 2:**
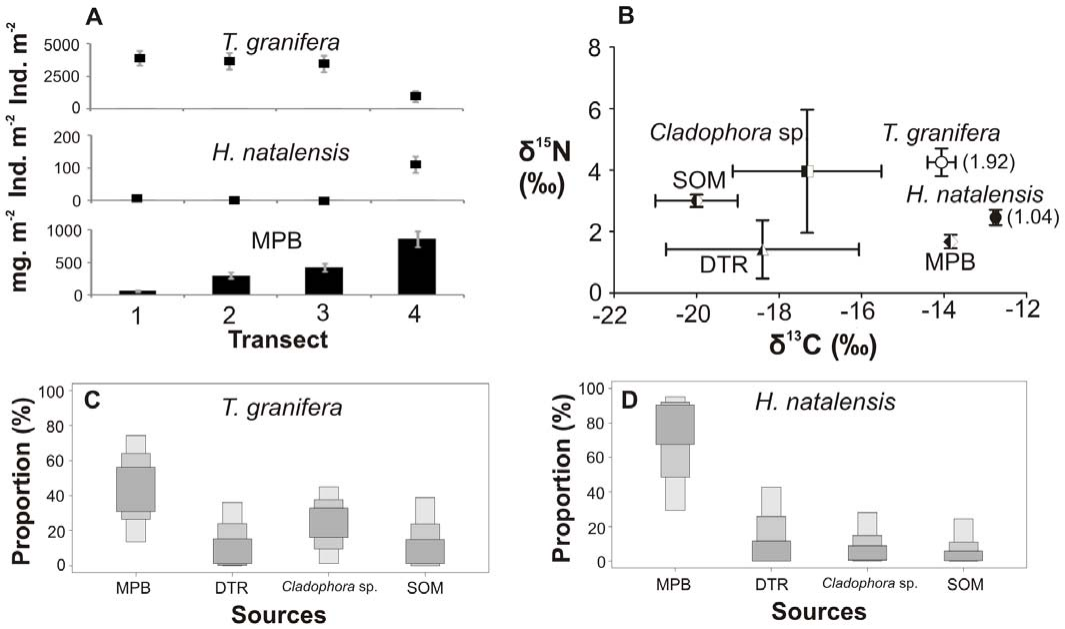
Gastropods, food sources and diets in Catalina Bay, 2007. (A) Four-point 6 m transect showing gastropod densities and microphytobenthic (MPB) biomass (as chl-*a* concentration). (B) Carbon and nitrogen stable isotope signatures of gastropods (number in brackets: trophic position) and their potential food sources; such as detritus (DTR) and sedimentary organic matter (SOM). SIAR boxplots show the proportional contribution (%) of different food sources to the diet of (C) *Tarebia granifera* and (D) *Haminoea natalensis*. Samples were collected in a freshwater seepage area of Catalina Bay in June 2007. Salinity ranged from 16 to 32. The water level of the South Lake rose due to the March 2007 mouth breach, after which the freshwater ponds associated with seepage areas along the eastern shores were flooded by seawater.

**Figure 3 pone-0031897-g003:**
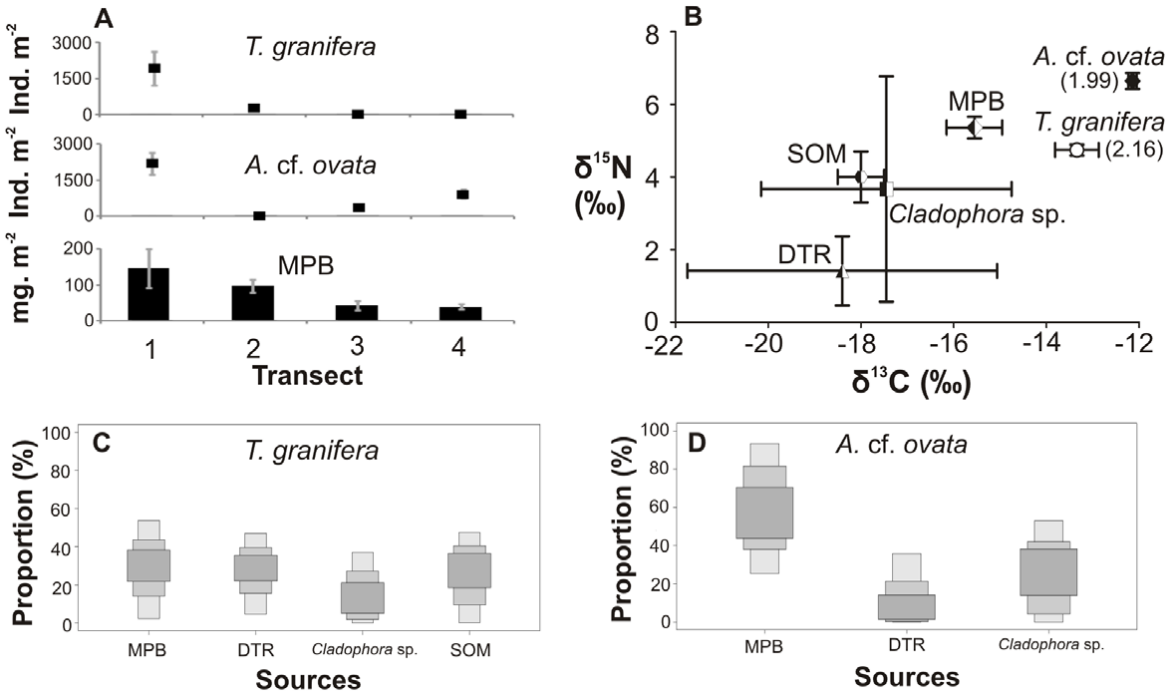
Gastropods, food sources and diets in Catalina Bay, 2009. (A) Four-point 6 m transect showing gastropod densities and microphytobenthic (MPB) biomass (as chl-*a* concentration). (B) Carbon and nitrogen stable isotope signatures of gastropods (number in brackets: trophic position) and their potential food sources, such as detritus (DTR) and sedimentary organic matter (SOM). SIAR boxplots show the proportional contribution (%) of different sources to the diet of (C) *Tarebia granifera*, (D) *Assiminea* cf. *ovata*. Samples were collected in a freshwater seepage area of Catalina Bay in October 2009. Salinity ranged from 1 to 10. The water level at South Lake was once again low, thereby allowing the formation of fresh and brackish water ponds along the eastern shores.

**Figure 4 pone-0031897-g004:**
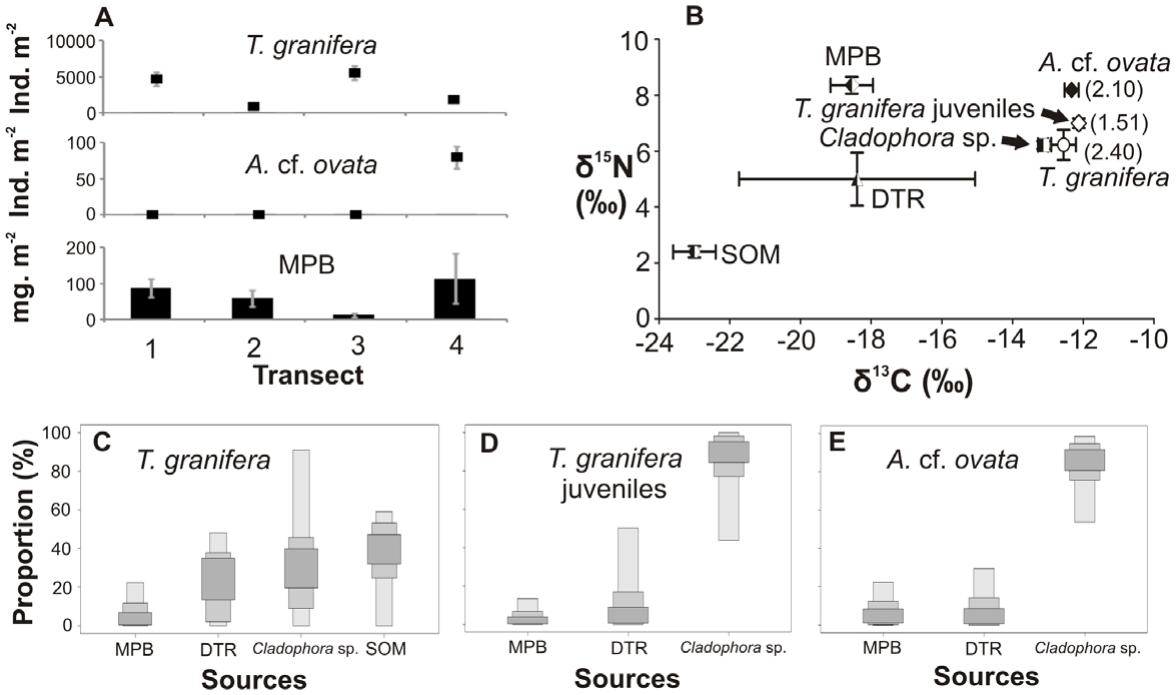
Gastropods, food sources and diets in Catalina Bay, 2010. (A) Four-point 10 m transect showing gastropod densities and microphytobenthic (MPB) biomass (as chl-*a* concentration). (B) Carbon and nitrogen stable isotope signatures of gastropods (number in brackets: trophic position) and their potential food sources, such as detritus (DTR) and sedimentary organic matter (SOM). SIAR boxplots show the proportional contribution (%) of different sources to the diet of (C) *Tarebia granifera* (shell height ≈13 mm), (D) *T. granifera* juveniles (shell height ≤5 mm) and (E) *Assiminea* cf. *ovata*. Samples were collected in a freshwater seepage area of Catalina Bay in February 2010. Salinity was 0.15–0.8. Although the salinity of the South Lake ranged between 47 and 56, its water level continued to decrease and larger freshwater ponds were formed in seepage areas along the eastern shores.

**Figure 5 pone-0031897-g005:**
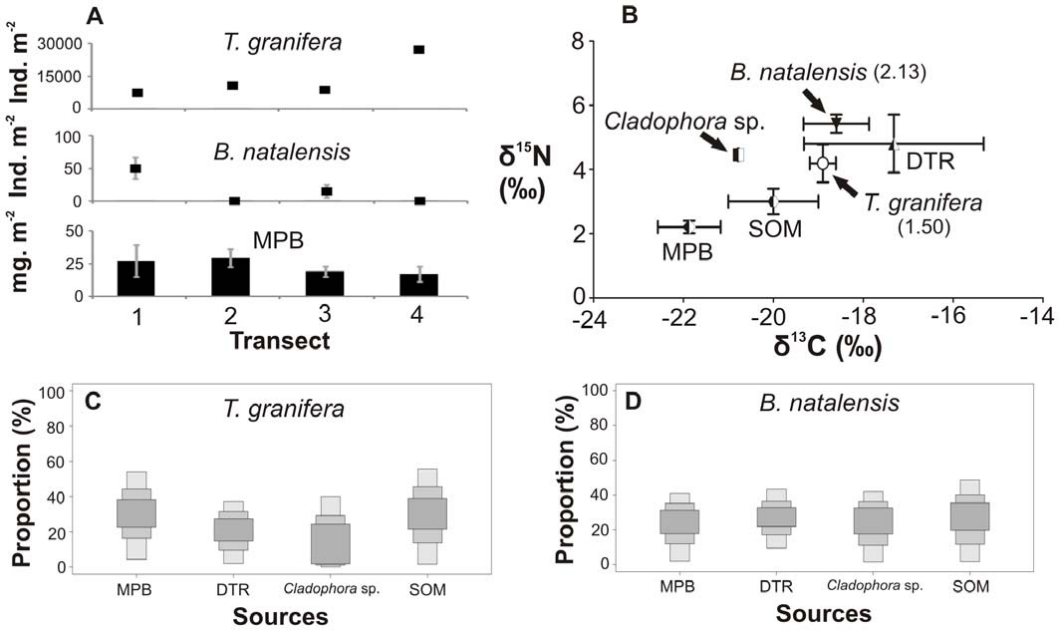
Gastropods, food types and diets in Lake Sibaya, 2009. (A) Four-point 3 m transect showing gastropod densities and microphytobenthic (MPB) biomass (as chl-*a* concentration). (B) Carbon and nitrogen stable isotope signatures of gastropods (number in brackets: trophic position) and their potential food sources, such as detritus (DTR) and sedimentary organic matter (SOM). SIAR boxplots show the proportional contribution (%) of different sources to the diet of (C) *Tarebia granifera* and (D) *Bulinus natalensis*. Samples were collected in November 2009. This lake has no connection to the sea and contains pure freshwater.

**Figure 6:**
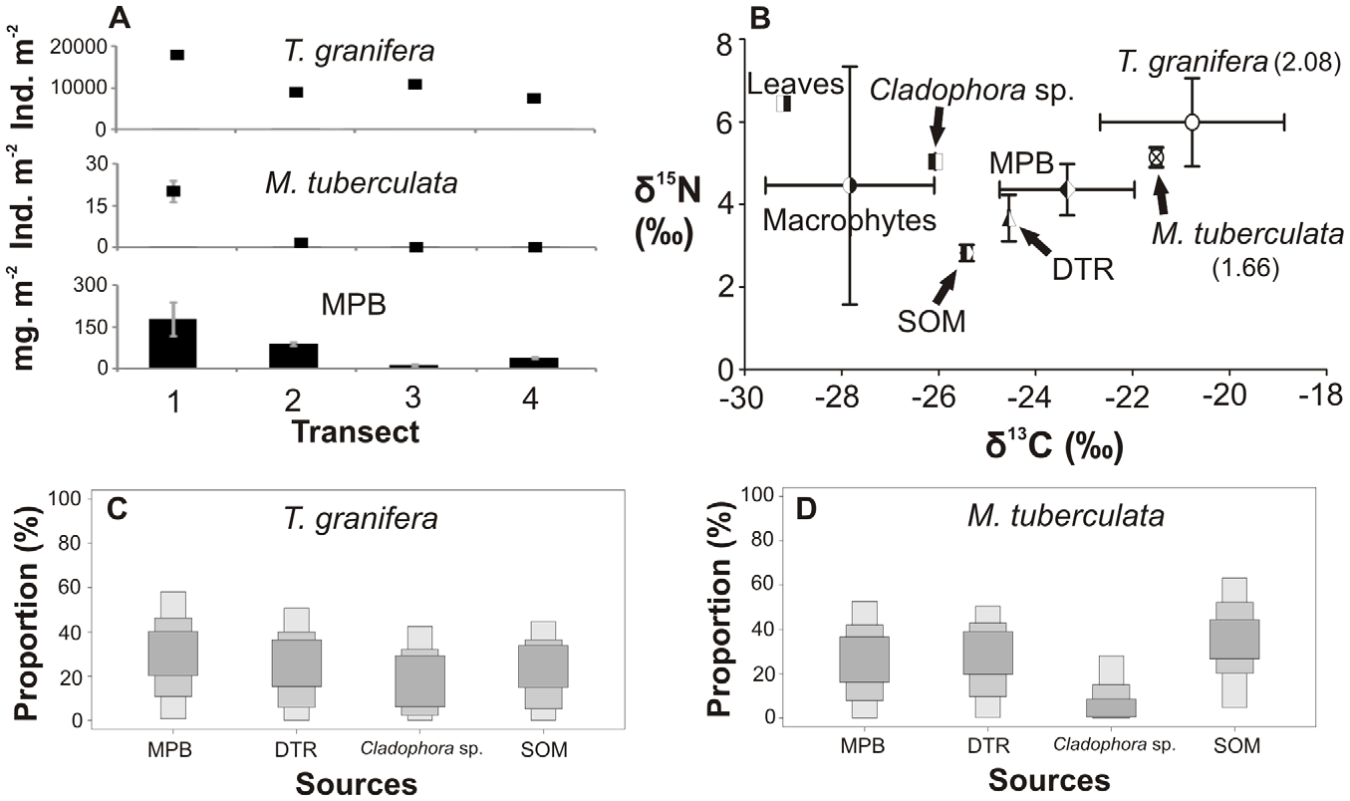
Gastropods, food sources and diets in Lake Nhlange, 2008. (A) Four-point 3 m transect showing gastropod densities and microphytobenthic (MPB) biomass (as chl-*a* concentration). (B) Carbon and nitrogen stable isotope signatures of gastropods (number in brackets: trophic position) and their potential food sources, such as detritus (DTR) and sedimentary organic matter (SOM). SIAR boxplots show the proportional contribution (%) of different sources to the diet of (C) *Tarebia granifera* and (D) *Melanoides tuberculata*. Samples were collected at Lake Nhlange in Kosi Bay, in November 2008. At the time, the site was dominated by freshwater conditions.

*Tarebia granifera* exhibited a patchy distribution and tended to be concentrated in shallow water (depth <1 m). Transects revealed spatial overlap between *T. granifera* and *Haminoea natalensis* (Fig. 2A), *Assiminea* cf. *ovata* (Fig. 3A and 4A), *Bulinus natalensis* (Fig. 5A) and *Melanoides tuberculata* (Fig. 6A) at respective sampling sites in the three lakes of Maputaland (Fig. 1). *Tarebia granifera* was found together with native gastropods in a total of 10 of the 20 transect points sampled. Of the 10 transect points where these gastropods were found together, 7 were at the edge of the *T. granifera* patch (transect points 1 and 4). *Haminoea natalensis* was the only native gastropod not to be found in the middle (transect points 2 and 3) of a *T. granifera* patch.

Gastropod densities as well as available microphytobenthic (MPB) biomass varied within transects (Fig. 2A–[Fig pone-0031897-g003] [Fig pone-0031897-g004] [Fig pone-0031897-g005] 6A; Table S1).Generally, native gastropods had lower population densities when compared to *T. granifera* (Table S1). However, *Assiminea* cf. *ovata* was found in densities greater than *T. granifera* at Catalina Bay in 2009 (Table S1). MPB biomass was available as a food source to gastropods at all sites, although its biomass was variable (Table S1). MPB biomass was not significantly correlated with gastropod densities (Table 1), despite being positively correlated with gastropod MPB diets (Table 1). Native gastropod MPB diet was strongly positively correlated with *T. granifera* MPB diet; however, it was negatively correlated with *T. granifera* density (Table 1).

**Table 1:** Correlations between microphytobenthic biomass and gastropod densities and diets.

		MPB	*T. granifera* density	Native density	*T. granifera* MPB diet	Native MPB diet
**MPB**	***r***	1	−0.211	−0.021	0.615	0.49
	**p**		ns	ns	<0.001	<0.05
***T. granifera*** ** density**	***r***	−0.211	1	−0.232	0.036	−0.330
	**p**	ns		ns	ns	<0.05
**Native density**	***r***	−0.021	−0.232	1	−0.114	0.354
	**p**	ns	ns		ns	<0.05
***T. granifera*** ** MPB diet**	***r***	0.615	0.036	−0.114	1	0.649
	**p**	<0.001	ns	ns		<0.001
**Native MPB diet**	***r***	0.409	−0.330	0.354	0.649	1
	**p**	<0.05	<0.05	<0.05	<0.001	

Pearson correlations (*r* is the correlation coefficient; *n* = 60 for every case) between microphytobenthic (MPB) biomass, *Tarebia granifera*, native gastropod densities and corresponding contribution of MPB to their diets. Diets were estimated by a stable isotope mixing model. Samples were collected at three coastal lakes in Maputaland. Native gastropods were *Haminoea natalensis*, *Assiminea* cf. *ovata*, *Bulinus natalensis* and *Melanoides tuberculata*.

### Gastropod diets

Gut content analysis revealed that microalgae and detritus were ingested by all gastropods (Table 2). Filamentous algae were also found in the guts of most gastropods (Table 2): their occurrence was 89.17% in *T. granifera* and 92% in native gastropods. Sand particles were only found in the guts of *H. natalensis*, *B. natalensis*, and in smaller amounts in *T. granifera* adults (shell height ≥10 mm) and *M. tuberculata* (Table 2). Distinctive remains of meiofauna were not found in gut contents. Macrophytes and fringing vegetation such as reeds, sedges and grasses, were excluded as potential food sources as their presence was not detected in the gut.

**Table 2:** Gastropod gut content analyses in three coastal lakes of Maputaland.

		Gut contents			
Location and year	Species	Microalgae	Detritus	Filamentous algae	Sand particles
CB 2007	*Tarebia granifera*	100	100	90	25
	*Haminoea natalensis*	100	100	100	90
CB 2009	*T. granifera*	100	100	75	15
	*Assiminea* cf. *ovata*	100	100	90	0
CB 2010	*T. granifera*	100	100	100	30
	*T. granifera* juveniles	100	100	100	0
	*A.* cf. *ovata*	100	100	100	0
LS 2009	*T. granifera*	100	100	80	5
	*Bulinus natalensis*	100	100	85	100
LN 2008	*T. granifera*	100	100	90	10
	*Melanoides tuberculata*	100	100	85	15

Gut contents were classified based on gross morphology. Data are presented as percentage occurrence in the guts of gastropods (n = 20). Samples were collected at Catalina Bay (CB) in the St. Lucia Estuary, Lake Sibaya (LS) and Lake Nhlange (LN) in Kosi Lakes.

Statistical analyses revealed that *T. granifera* δ^13^C and δ^15^N signatures differed significantly between locations and between times at Catalina Bay (Table 3, Fig. 2B–[Fig pone-0031897-g003] [Fig pone-0031897-g004] [Fig pone-0031897-g005] 6B). The δ^13^C signature of food source types also differed significantly between locations and between times (Table 4, Fig. 2B–[Fig pone-0031897-g003] [Fig pone-0031897-g004] [Fig pone-0031897-g005] 6B). The δ^15^N signature of food source types did not differ significantly between locations (Table 4), although it differed significantly over time at Catalina Bay (Table 4). There was no significant difference between the trophic position of *T. granifera* and that of native gastropods (t-test, df = 8, t = 0.406, p>0.05).

**Table 3:** Statistical analyses of stable isotope signatures of *Tarebia granifera*.

Dependant variable	Source	df	MS	F	p
**δ^13^C**	Location	2	136.126	118.7	<0.001
	Error	22	1.147		
	Time	2	4.324	34.538	<0.001
	Error	12	0.125		
**δ^15^N**	Location	2	4.052	5.07	<0.05
	Error	22	0.799		
	Time	2	5.074	44.337	<0.001
	Error	12	0.114		

ANOVA for δ^13^C and δ^15^N of *T. granifera* from Catalina Bay, Lake Sibaya and Lake Nhlange (different locations in Maputaland), and from 2007, 2009 and 2010 at Catalina Bay (different times).

**Table 4:** Statistical analyses of stable isotope signatures of gastropod food source types.

Dependant variable	Source	df	MS	F	p
**δ^13^C**	Food×Location	6	17.079	4.381	<0.05
	Error	48	3.899		
	Food×Time	6	16.138	5.519	<0.05
	Error	24	2.924		
**δ^15^N**	Food×Location	6	4.659	1.477	ns
	Error	48	3.155		
	Food×Time	6	8.881	6.356	<0.001
	Error	24	1.397		

Two-way ANOVA for δ^13^C and δ^15^N, including microphytobenthos, detritus, *Cladophora* sp. and sedimentary organic matter (different food source types) collected at Catalina Bay, Lake Sibaya and Lake Nhlange (different locations in Maputaland), and collected in 2007, 2009 and 2010 at Catalina Bay (different times).

Stable isotope mixing model results indicated the largest proportional contributions of food sources to the diets of gastropods (Fig. 2C–[Fig pone-0031897-g003] [Fig pone-0031897-g004] [Fig pone-0031897-g005] 6C, Table S1). At Catalina Bay, in 2007 microphytobenthos (MPB) made the greatest contribution to the diet of *T. granifera* (13–74% of total); in 2009 it was MPB (2–53%), detritus (4–46%) and sedimentary organic matter (SOM) (0–47%); and in 2010 *Cladophora* sp. (0–61%) and SOM (0–58%). At Lake Sibaya, MPB (4–54%) and SOM (1–55%) made the greatest contribution to the diet of *T. granifera*; but at Lake Nhlange it was MPB (0–57%) and detritus (0–505) (Fig. 2C–[Fig pone-0031897-g003] [Fig pone-0031897-g004] [Fig pone-0031897-g005] 6C, Table S1). *Cladophora* sp. (43–93%) made the greatest contribution to the diet of *T. granifera* juveniles at Catalina Bay in 2010 (Fig. 4D, Table S1 *). Haminoea natalensis* fed on MPB (29–95%) (Fig. 2D, Table S1). *Assiminea* cf. *ovata* fed on MPB in 2009 (25–93%) and *Cladophora* sp. in 2010 (53–98%) (Fig. 3D, Fig. 4E, Table S1). At Lake Sibaya, the native *B. natalensis* appeared to feed on a combination of all available food sources (Fig. 5D, Table S1). At Lake Nhlange, *M. tuberculata* diet consisted for the most part of MPB (0–55%), detritus (DTR) (0–50%) and SOM (4–63%) (Fig. 6D, Table S1).

Assuming that the microalgae found in the gut content analysis correspond to the mixing model MPB food source, detritus to DTR and filamentous algae to *Cladophora* sp.; there was a significant correlation between gut contents and diet estimated from stable isotope mixing models (n = 31, r^2^ = 0.69, p = 0.139).

Isotopic (stable isotope) dietary overlap (IDO) was significant (over 60%) between *T. granifera* and native gastropods in most cases (Table 5). The IDO between *T. granifera* adults and *A.* cf. *ovata* was not significant (below 60%) at Catalina Bay in 2010 (Table 5). The IDO between *T. granifera* adults and juveniles was also not significant (Table 5). However, the IDO of 95% between *T. granifera* juveniles (shell height ≤5 mm) and *A.* cf. *ovata* was the highest recorded in the study (Table 5).

**Table 5:** Isotopic (stable isotopes) dietary overlap between gastropods in three coastal lakes of Maputaland.

Gastropod species	Location and year	Salinity	IDO (%)
*Tarebia granifera* a and *Haminoea natalensis*	CB 2007	16–32	75
*T. granifera* and *Assiminea* cf. *ovata*	CB 2009	1–10	62
*T. granifera* and *A.* cf. *ovata*	CB 2010	0.15–0.8	50
*T. granifera* juveniles^a^ and *A.* cf. *ovata*	CB 2010	0.15–0.8	95
*T. granifera* and *T. granifera* juveniles	CB 2010	0.15–0.8	49
*T. granifera* and *Bulinus natalensis*	LS 2009	0.18–0.45	87
*T. granifera* and *Melanoides tuberculata*	LN 2008	0.91–1.45	85

a *T. granifera* = shell height ≥10 mm; *T. granifera* juveniles = shell height ≤5 mm; all other gastropods were adults.

Locations are Catalina Bay (CB) in the St. Lucia Estuary, Lake Sibaya (LS) and Lake Nhlange (LN) in Kosi Lakes. Isotopic dietary overlap (IDO, %) values over 60% indicate significant overlap and a value of 100% indicates absolute overlap.

## Discussion

According to the competitive exclusion principle, species with identical ecological niche compete with each other for resources and, if these are limiting, there is a tendency towards the exclusion of the weaker competitor or the development of resource partitioning or niche differentiation [59]–[61]. Although recent research indicates that competition involving invasive species only rarely causes local extinction [62], there is still a need to better understand the mechanisms of competitive interactions and how the ecosystem is affected.

This study focuses only on situations where spatial overlap existed between the invasive gastropod *Tarebia granifera* and another single native gastropod species. Yet the spatial distribution of *T. granifera* can simultaneously overlap with that of several species at a time [26]. However, this study does not address the greater complexity of interactions between multiple gastropod species in the same area.

*Tarebia granifera* invaded the shallow waters (less than 2 m depth) of Kosi Bay's Lake Nhlange, Lake Sibaya and the eastern shores of the South Lake of the St. Lucia Estuary (Fig. 1). The *T. granifera* populations tend to form dense patches (often >1000 ind. m^−2^) of varying dimensions. *Tarebia granifera* patches such as the ones sampled in this study are often found in very shallow embayments with clear sandy substrate where particulate organic matter settles or where there is freshwater seepage. In contrast, the native species in this study have much lower population densities and their distribution is more extensive and much less patchy [26]. E.g. *Assiminea* cf. *natalensis* at the St. Lucia Estuary is a historically dominant species found throughout the system [35]. *Tarebia granifera* also appears to be displacing native gastropods from certain areas in the iSimangaliso Wetland Park (Fig. 1), whereas the native species do not appear to be mutually exclusive.

*Tarebia granifera* has previously been classified as both detritivore and grazer [31], [63]. This study shows that *T. granifera* has a generalist diet which includes a wide variety of food sources. *Tarebia granifera* is established in all major coastal lakes of Maputaland (Fig. 1), where in particular cases its diet was either largely composed of a single or a combination of general food sources (Fig. 2C–[Fig pone-0031897-g003] [Fig pone-0031897-g004] [Fig pone-0031897-g005] 6C, Table S1). Native gastropods *A.* cf. *ovata* and *Haminoea natalensis* tended to favor a single food source in Catalina Bay, whereas *Bulinus natalensis* and *Melanoides tuberculata* had a more varied diet in Lake Sibaya and Lake Nhlange respectively. Microphytobenthos (MPB) featured in the diet of all gastropods and its proportional contribution was always positively correlated with its available biomass in the environment (Table 1). Although *T. granifera* in high densities had the potential to consume MPB until it became limiting [31], there were other food sources available to gastropods, such as detritus (DTR), the filamentous algae *Cladophora* sp. and sedimentary organic matter (SOM) (Table S1).

Autochthonous primary production (represented by MPB, *Cladophora* sp. and SOM) contributed on average 78% of gastropod diets. δ^13^C signatures were significantly more enriched at Catalina Bay, when compared with Lake Nhlange and Lake Sibaya, which is expected because of the influence of salt water at that site [64]. Detritus (DTR) is the only food source that could represent an allochthonous contribution in the form of terrestrial plant matter, and it only contributed on average up to 20% of gastropod diets. Although terrestrial primary production can support secondary production of aquatic mollusks (since most species, including *T. granifera*, have the ability to feed on [65] and digest terrestrial plant matter via cellulase activity [66]), aquatic primary production was more important for the gastropods at the coastal lakes in this study. Animal contributions, in the form of decaying matter which would not be easily recognized in a gut content analysis, can also be represented in the DTR signature which would explain its wide variation in δ^13^C. Decaying animal matter and/or meiofaunal organisms can make a small but significant contribution to the diet of *T. granifera*. *Melanoides tuberculata*, which in this study exhibited a diet identical to that of *T. granifera*, is capable of assimilating animal matter [53]. This opens the possibility that *T. granifera* may also feed on dead gastropods, which would be beneficial to its survival at very high densities and under unfavorable environmental conditions. Detritus and sedimentary organic matter may account for the high organic carbon content (up to 60%) not accounted for by the gut fluorescence technique in *T. granifera* [31]. However, although as many samples as possible were collected and broadly classified, due to logistical limitations not all potential food sources may have been considered in this study.

*Assiminea* cf. *ovata* has an affinity for MPB, as do other assimineid species [67]. However, the increase in abundance of *T. granifera* at St Lucia from 2009 to 2010 may have influenced a dietary shift in *A.* cf. *ovata*, which started to feed less on MPB and more on *Cladophora* sp. (Fig. 3D, 4E and Table S1). This is not surprising, as dietary shifts in native species tend to be caused by non-native species, as they disrupt trophic connections [1]. If resource partitioning between *T. granifera* and *A.* cf. *ovata* took place, it was effective in reducing IDO from 62% in 2009 to 50% in 2010. However, it is very likely that dietary shifts occured in response to changing environmental conditions, which affected food availability and quality at Catalina Bay.

Due to its remarkable salinity tolerance, *T. granifera* managed to survive the unusual 2007 mouth breaching event when seawater entered the St. Lucia Estuary and flooded the freshwater seepage areas of Catalina Bay (Fig. 1) [30]. *Assiminea* cf. *ovata* was abundant in most of the seepage areas only in 2008, while the salinity level was high (around 30) [26]. When fresh water conditions became re-established in the seepage areas in 2009, *T. granifera* increased in numbers and spread until 2010, when it once again dominated benthic assemblages whilst evidently displacing *A.* cf. *ovata* [26]. Along the eastern shores of South Lake (Fig. 1), *A.* cf. *ovata* continued to be abundant in areas with high salinity and was even found in a few freshwater seepage areas where *T. granifera* was not present (e.g. Dead Tree Bay: 28°9′2.83″S, 32°27′53.76″E). At least over the last six years, *A.* cf. *ovata* and *H. natalensis* have consistently been found together in shallow-water habitats on the western shores of South Lake (Fig. 1), where there are fewer freshwater seepage areas, with only one being found to harbor *T. granifera*, i.e. Makakatana Bay (28°14′10.87″S, 32°25′11.61″E). In contrast to *T. granifera*, *H. natalensis* does not appear to displace *A.* c.f. *ovata*.

Salinity is indeed one of the most important factors influencing the distribution of gastropods in coastal lakes of Maputaland [26]. Although there was a significant IDO between *T. granifera* and *H. natalensis*, there was very little spatial overlap between the two, since *H. natalensis* preferred the saltier conditions outside the seepage area. High salinity conditions were also associated with *A.* cf. *ovata* [26]. When salinity dropped below 20, *T. granifera* expanded its range and density. *Assiminea* cf. *ovata* was recorded in the St. Lucia Estuary at low salinities, but only when *T. granifera* was either absent or present in relatively low densities. This suggests that *T. granifera* may displace *A.* cf. *ovata* only under specific salinity conditions. This also supports the theory that vulnerable native species can resist invasion by non-native species under certain conditions [68].

Interestingly, at Catalina Bay in 2010, it was observed that both *A.* cf. *ovata* and *T. granifera* juveniles tended to concentrate in extremely shallow areas (depth <0.1 metre), whereas *T. granifera* adults tended to concentrate in adjacent deeper waters (depth >0.1 metre). In this environment, where water level fluctuation is commonplace, it is conceiveable that smaller snails would continue to feed in extremely shallow areas, whereas larger snails tend to move away and/or burrow to avoid dessication. This behavior reduces the potential for competitive interactions involving *T. granifera* adults and can be interpreted in terms of spatial niche differentiation. However, the spatial overlap between *A.* cf. *ovata* and *T. granifera* juveniles remains unchanged and becomes effectively greater than that between *A*. cf. *ovata* and *T. granifera* adults. *Tarebia granifera* juveniles are often numerous, have fast growth rates and may even grow faster and larger in the presence of other snails [24], [31], [69]. They are voracious feeders and may have greater impacts on food stocks than adults [31], [70]. It is therefore suggested that *T. granifera* juveniles played a role in the displacement of *A.* cf. *ovata*. A high degree of spatial overlap between *T. granifera* adults and juveniles has been recorded [26], however, there is also evidence of an ontogenetic dietary shift in *T. granifera*, since SOM was only used by adults and IDO was not significant (Table 5, Table S1, Fig. 4C–D). Resource partitioning certainly minimizes potential competition between *T. granifera* adults and juveniles.

Food resource use within populations at the microhabitat scale can be a significant factor in gastropod interactions [71]. Some of the large variations in isotopic signatures recorded for gastropods in this study may be due to individual variation, which in turn may be primarily affected by habitat heterogeneity and resource availability [71]. This adds to the complexity of addressing dietary overlap and potential competition for food resources between gastropods.

*Tarebia granifera* and *B. natalensis* appear to have very similar and overlapping diets (Table S1, Table 5). Thiarids and sympatric pulmonates can have similar diets [72]. The lack of competitive exclusion may be explained by selective ingestion and behavioral differences [73], as well as selection of different micro-habitats, such as macrophyte fronds where *B. natalensis* positions itself close to the water surface [74], thus avoiding physical interactions with *T. granifera* which tend to stay on the substrate. However, in the Caribbean, *T. granifera* is known to displace *Biomphalaria glabrata* (Say, 1818) (pulmonate, Planorbidae), which like *B. natalensis* is associated with submerged vegetation rather than the substrate. Further empirical studies are thus needed to address interactions between *T. granifera* and *B. natalensis* which may involve chemical cues rather than competition for food [27], [75].

*Tarebia granifera* has been widely reported to out-compete *M. tuberculata* for food resources and space and there is concern that other native thiarids, such as *Thiara amarula* (Linnaeus, 1758) may also be negatively affected [24]. As expected, the diets of these gastropods are similar and IDO is significant (Table 5, Table S1), suggesting that *T. granifera* and *M. tuberculata* can compete for resources. Yet, there is spatial overlap between these species in Lake Nhlange. This apparent competitive co-existence [76] may be explained by the generalist diet of both species together with adequate availability of resources.

At Catalina Bay and Lake Nhlange, there was no indication that food resources were limiting. The lowest available MPB biomass was recorded on the eastern shores of Lake Sibaya in a sheltered bay (table S1). A larger proportion of the eastern shore of Lake Sibaya is comprised of exposed sandy terraces that are even poorer in nutrients in comparison to sheltered bays, which tend to accumulate organic deposits [77]. *Tarebia granifera* is currently the only recorded gastropod species dominating the benthic assemblage in open terraces of Lake Sibaya [26]. The unusually small size of the *T. granifera* individuals at Lake Sibaya's open terraces may be explained by lower food availability and quality [26]. This suggests that food resources are limiting in large areas of Lake Sibaya and this may have played a role in the apparent displacement of the historically abundant native gastropods *M. tuberculata* and *Bellamya capillata* (prosobranch, Viviparidae) [34], [78], by *T. granifera*. However, both *M. tuberculata* and *B. capillata* may still persist in parts of Lake Sibaya that have not been surveyed and particularly at depths greater that 5 m where less *T. granifera* can be found.

In the St. Lucia Estuary, there appears to be a relatively high concentration of dissolved nutrients despite the drought conditions [79], [80]. Nutrient input is likely to come from fecal matter of ungulates, waterfowl and particularly hippopotami that frequent freshwater seepage areas and play a part in shaping them [81], [82]. Nutrient input and concentrations are also affected by water level fluctuations which can be extremely variable at Catalina Bay. This may explain the δ^15^N signature variation and enrichment observed at Catalina Bay in 2010. Nutrient enrichment certainly led to the increase in abundance of *Cladophora* sp., which featured prominently in gastropod diets at Catalina Bay only in 2010. Nutrient enrichment can have overriding effects on several system processes [83], initially favoring both native and non-native species. However, *T. granifera* appear to have a greater and faster growth per unit resource consumption, coupled with earlier onset of reproduction, which contributes to their invasion success [84], [85].

Although in the same trophic level, and thus probably performing a similar ecosystem function, *T. granifera* differs from the native benthic invertebrates of Maputaland in many ways. Besides having different physiology, behavior and life history, *T. granifera* can also differ from other species in terms of its elemental turnover rate [86] and biomass accrual. *Tarebia granifera* populations attain extremely high densities due to their high parthenogenetic reproduction rate. Large amounts of carbon are therefore sequestered by *T. granifera*. The average size of individuals within gastropod species is naturally a factor in determining the biomass of the different populations. When compared to native gastropod species, such as *B. capillata* at Lake Sibaya, the estimated per capita biomass of *T. granifera* can be on average ten times lower (unpublished data). At Catalina Bay, the average per capita biomass of *T. granifera* is about double that of *A.* cf. *ovata* (unpublished data). However, the biomass of *T. granifera* populations is often much greater than that of potentially vulnerable native gastropods in the same trophic level. Therefore, the invasion of sites such as Catalina Bay by *T. granifera* results in the sequestration of a lot more carbon than usual in benthic primary consumers. This might not be an issue in terms of reducing the availability of nutrients to native gastropods, since there seem to be a great abundance of nutrient resources at Catalina Bay. At sites like Lake Sibaya, the invasion of *T. granifera* may not result in the sequestration of such high magnitudes of carbon, but because nutrients can be limiting, *T. granifera* can at least affect their availability to native gastropods and interfere with vital nutrient cycling dynamics in complex ways [31], [87]–[89].This study did not focus on higher trophic level interactions. Indeed, there is a general lack of multiple trophic level studies that may reveal more complex ecological responses [90]. However, snails like *T. granifera* do not seem to be successfully predated upon by native species in iSimangaliso, although they can be attacked by birds, fish and invertebrates, such as crabs and leeches. Trematode parasites are also absent in *T. granifera* [26]. Predators and parasites could regulate populations of alien gastropods [45]. The lack of successful predation and parasitism is thus likely to facilitate the establishment and spread of *T. granifera* in South Africa.

## Supporting Information

Table S1 Available microphytobenthos biomass and gastropod densities and diets in three coastal lakes of Maputaland. The contributions of microphytobenthos (MPB), detritus (DTR), *Cladophora* sp. and sedimentary organic matter (SOM) to the diets of gastropods were calculated with the SIAR (stable isotope analysis in R) mixing model using Carbon (δ^13^C) and Nitrogen (δ^15^N) signatures. Gastropod shell height (range) was recorded. Available MPB biomass is presented as pigment concentration.
(DOC)
