# Supplementary material for: Stable Isotope Evidence for Dietary Overlap between Alien and Native Gastropods in Coastal Lakes of Northern KwaZulu-Natal, South Africa
Source: PLoS One. 2012 Feb 21;7(2):e31897. doi: 10.1371/journal.pone.0031897 (PMC3283693; doi:10.1371/journal.pone.0031897)
Supplement: Table S1 — Available microphytobenthos biomass and gastropod densities and diets in three coastal lakes of Maputaland. The contributions of microphytobenthos (MPB), detritus (DTR), Cladophora sp. and sedimentary organic matter (SOM) to the diets of gastropods were calculated with the SIAR (stable isotope analysis in R) mixing model using Carbon (δ13C) and Nitrogen (δ15N) signatures. Gastropod shell height (range) was recorded. Available MPB biomass is presented as pigment concentration. (DOC) [file pone.0031897.s001.doc]

**Table S1. Available microphytobenthos biomass and gastropod densities and diets in three coastal lakes of Maputaland.** The contributions of microphytobenthos (MPB), detritus (DTR), *Cladophora* sp. and sedimentary organic matter (SOM) to the diets of gastropods were calculated with the SIAR (stable isotope analysis in R) mixing model using Carbon (δ^13^C) and Nitrogen (δ^15^N) signatures. Gastropod shell height (range) was recorded. Available MPB biomass is presented as pigment concentration.

|  |  |  |  |  | **Contribution to diet (95 % credible interval range and {mean})** | | | |
| --- | --- | --- | --- | --- | --- | --- | --- | --- |
| **Location and year** | **Mean MPB biomass ± SD**  **(mg. m^-2^)** | **Gastropod species** | **Shell height (mm)** | **Mean density ± SD (ind. m^-2^)** | **MPB** | **DTR** | ***Cladophora* sp.** | **SOM** |
| Catalina Bay 2007 | 412.92 ± 51.64 | *Haminoea natalensis* | 10 - 22 | 22 ± 8 | 29 – 95 {65} | 0 – 42 {15} | 0 - 28 {10} | 0 - 24 {7} |
|  |  | *Tarebia granifera* | 8 -12 | 2745 ± 441 | 13 - 74 {43} | 0 - 36 {15} | 1 - 44 {24} | 0 - 38 {16} |
| Catalina Bay 2009 | 76.17 ± 14.16 | *Assiminea* cf. *ovata* | 2-5 | 819 ± 117 | 25 - 93 {58} | 0 - 35 {14} | 0 - 53 {26} | 0 {0} |
|  |  | *T. granifera* | 13-29 | 425 ± 111 | 2 - 53 {30} | 4 - 46 {27} | 0 - 37 {18} | 0 - 47 {25} |
| Catalina Bay 2010 | 66.56 ± 7.25 | *A.* cf. *ovata* | 2-5 | 14 ± 4 | 0 - 22 {8} | 0 - 29 {10} | 53 - 98 {80} | 0 {0} |
|  |  | *T. granifera* juveniles | 1-5 | 1662 ± 143 | 0 - 13 {4} | 0 - 35 {13} | 43 - 93 {81} | 0 {0} |
|  |  | *T. granifera* | 10-28 | 818 ± 70 | 0 - 22 {8} | 0 - 47 {24} | 0 – 61 {32} | 0 - 58 {35} |
| Lake Sibaya 2009 | 23.83 ± 3.09 | *Bulinus natalensis* | 3-7 | 14 ± 2 | 2 - 41 {23} | 9 - 43 {26} | 1 - 42 {23} | 1 - 48 {26} |
|  |  | *T. granifera* | 5-16 | 10842 ± 784 | 4 - 54 {30} | 1 - 37 {20} | 0 - 39 {18} | 1 - 55 {30} |
| Lake Nhlange 2008 | 79.58 ± 17.98 | *Melanoides tuberculata* | 7-18 | 5 ± 1 | 0 - 52 {27} | 0 - 50 {27} | 0 - 28 {10} | 4 - 63 {35} |
|  |  | *T. granifera* | 6-11 | 9871 ± 290 | 0 - 57 {30} | 0 - 50 {25} | 0 - 42 {20} | 0 - 44 {23} |
